# What is best for Esther? A simple question that moves mindsets and improves care

**DOI:** 10.1186/s12913-023-09870-1

**Published:** 2023-08-17

**Authors:** Nicoline Vackerberg, Ann- Christine Andersson, Anette Peterson, Anette Karltun

**Affiliations:** 1 Jönköping Academy for Improvement of Health and Welfare, School of Health and Welfare, Jönköping University, Jönköping, Sweden; 2 Region Jönköping County, Jönköping, Sweden; 3 Department of Supply Chain and Operations Management, School of Engineering, Jönköping University, Jönköping, Sweden

**Keywords:** System-thinking, Complex care, Quality improvement, Person centeredness, Co-production, Collaboration, Perseverance, Mindset

## Abstract

**Background:**

Persons in need of services from different care providers in the health and welfare system often struggle when navigating between them. Connecting and coordinating different health and welfare providers is a common challenge for all involved. This study presents a long-term regional empirical example from Sweden—ESTHER, which has lasted for more than two decades—to show how some of those challenges could be met. The purpose of the study was to increase the understanding of how several care providers together could succeed in improving care by transforming a concept into daily practice, thus contributing with practical implications for other health and welfare contexts.

**Methods:**

The study is a retrospective longitudinal case study with a qualitative mixed-methods approach. Individual interviews and focus groups were performed with staff members and persons in need of care, and document analyses were conducted. The data covers experiences from 1995 to 2020, analyzed using an open inductive thematic analysis.

**Results:**

This study shows how co-production and person-centeredness could improve care for persons with multiple care needs involving more than one care provider through a well-established Quality Improvement strategy. Perseverance from a project to a mindset was shaped by promoting systems thinking in daily work and embracing the psychology of change during multidisciplinary, boundary-spanning improvement dialogues. Important areas were Incentives, Work in practice, and Integration, expressed through trust in frontline staff, simple rules, and continuous support from senior managers. A continuous learning approach including the development of local improvement coaches and co-production of care consolidated the integration in daily work.

**Conclusions:**

The development was facilitated by a simple question: “What is best for Esther?” This question unified people, flattened the hierarchy, and reminded all care providers why they needed to improve together. Continuously focusing on and co-producing with the person in need of care strengthened the concept. Important was engaging the people who know the most—frontline staff and persons in need of care—in combination with permissive leadership and embracing quality improvement dimensions. Those insights can be useful in other health and welfare settings wanting to improve care involving several care providers.

## Introduction


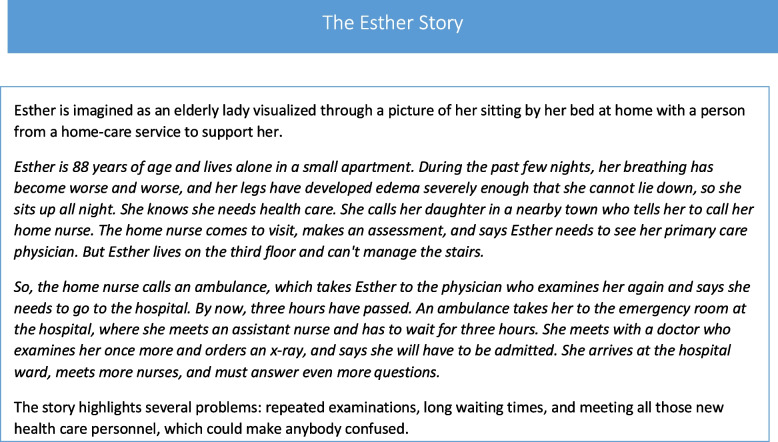


Source: Region Jönköping County, used with permission.

For persons in need of care from several providers in the health and welfare system, navigating between them is often a struggle. This is largely due to a lack of adequate coordination between different care providers in the hospital-, primary- and home care services related to different steering mechanisms such as funding, laws, strategies, regulations, and organizational boundaries [1, 2]. What is a clear and known routine for one care provider, for example when it comes to how to treat heart failure, may be different for another. When different care providers interconnect and there is a need for continued treatment, as for instance with chronic diseases, there may potentially be problems for the patient, including the treatment being interrupted. This interruption can negatively affect the well-being and recovery of the person in need of care. Connecting care providers in a satisfactory manner is a common challenge [2–6]. This study presents a long-term empirical example from Sweden on how these challenges could be managed.

In Sweden, care is mainly provided by the national public sector, which is divided into 21 regions and 290 municipalities with taxation power and a high degree of autonomy. The regions primarily provide hospital care, primary care, and advanced home health care, and the municipalities provide social care, home care, group housing, elder care, and daycare. The funding for hospital care is based on the level and extent of care provided, rather than being related to the number of beds. The calculation of the funding is based on the history from the previous years and indexed for the next year.

In the late 1990s, a system-wide health care project started in the Highland area in southern Sweden to redefine the care experience for the persons in need of continuous care from multiple care providers [7]. The area by then included 120 000 inhabitants and was characterized by having a larger percentage of elderly people [8]. This project aimed to deliver more person-centered care across the continuum of primary, hospital, and municipal care services. To highlight this, the project got the name of a person, namely *Esther*. The idea was that effective and efficient in- and outpatient care processes would be able to bridge gaps between different care providers who in the future would coordinate and deliver care based on the needs and preferences of “Esther” [7, 9, 10].

The name of the project was chosen based on the story of Esther and what happened when she got sick. The project used this story to shift the focus from the perspective of each care provider to that of the person (Esther) and what is important for her.

The following textbox clarifies some of the terms used in this paper.

**Table:** 

**Esther**	Person in need of care from more than one provider
**ESTHER**	
The project	The initial project period of two years, 1997–1999
The network	The years following after the project period, 1999–2016
The concept	The way of working today, integrated into everyday activities, from 2016 - 2020
The coaches	Health and social care professionals who, besides their normal work, are trained as improvement coaches to initiate and support organizational improvements with the aim of making care better for Esther, from 2006 - 2020

The ESTHER project was initiated in 1997 by the medical ward clinic at the Highland Hospital in the Region Jönköping County in cooperation with six Highland municipalities and primary care services in the area. While ESTHER started in elder care, representing a person with complex care needs who requires coordination and integration between the hospital, primary care, home care, and municipal care, Esther today represents a person of any age with complex care needs. This is because good collaboration and coordination should include a holistic view of any person’s health conditions and needs, regardless of age or diagnoses.

The Region Jönköping County uses “Quality as a strategy” as a basis for its development [7, 11, 12]. The ESTHER project, which later transitioned into a network and mindset, was regularly evaluated [10, 13]. It also received several awards for its innovative way of thinking by prioritizing the person´s needs, and has since spread to different parts of the world including Singapore, the UK, Denmark, and Austria. The international dissemination of ESTHER is at the time of writing studied by researchers at McMaster University, Canada, and Jönköping University.

This study focuses on the beginning of ESTHER in Sweden and how the work processes were developed, which had many effects. For example, the new work processes resulted in a decrease in hospital care and an increase of home care as the hospital could close more than half of its number of beds thanks to developing the support for local homecare; they also led to a high national ranking of patient satisfaction, confidence in the regional health care, and an empowered ESTHER and frontline staff who were enabled to co-produce improvement work. This became possible mainly owing to the development of a shared mental model among professionals, placing the person in need of care at the center [1]. The staff experience, the reasoning behind decisions made, and lessons learned over the course of two decades have not been made explicit before. This is presented in this paper as we suggest it could contribute to valuable knowledge on how to move mindsets that connect and improve care, which can inform and guide practice in other health and welfare contexts.

## Theoretical background

The overall goal of the ESTHER project was to develop a process-oriented and more holistic and person-centered system of health and welfare, constituted by local care providers, to achieve a better quality of care for Esther [10].

### Person-centered care

There are several definitions of person-centered care (PPC). In general, it means a shift from focusing on the disease to the person's focus [14, 15]. In this study, the principles described by Håkansson Eklund et al. [15] are guiding the work, by using the Esther concept, which includes a holistic approach where the needs and preferences of the individuals guide the care process [16, 17].

A person-centered way of working necessitates multi-professional and boundary-spanning organizational collaboration [17]. The team supporting Esther is responsible for collaborating and co-producing conditions that meet the individual’s needs [18]. Person-centeredness can also support autonomy and improve the person’s status [18, 19]. Person-centeredness further requires that the entire health and welfare system co-produces [20] a holistic system-wide work process.

Since this is what characterizes ESTHER, it can be considered PPC.

### Quality improvement

To achieve the overall goal of ESTHER, Quality Improvement (QI) knowledge was used and developed in practice. QI is a learning-driven process that includes dimensions such as systems thinking and the use of tools and measurements to understand variation and plan improvements, together with knowledge of the psychology of change, to improve systems performance and professional competencies [21, 22]. The Health Foundation [23] describes QI as

“*giving the people closest to issues affecting care quality the time, permission, skills, and resources they need to solve them. It involves a systematic and coordinated approach to solving a problem using specific methods and tools with the aim of bringing about a measurable improvement”( p.3).*

This specific QI competence was lacking in Health and Social care educational programs and therefore in need to be trained in practice. The ESTHER project was an example how to do this successfully [24].

In an evaluation of the ESTHER project by Erlandsson in 2001 [10], it was concluded that for process orientation to have sufficient impact, it is necessary to develop systems view among management and employees to create an understanding of other care providers in the care process. A process is defined as several activities that together produce something of value to users [25]. A basic assumption in quality management is that an organization can best be understood through its processes [26].

Considering these factors, developing care requires an ability to handle several challenges at once, given the complexity of health and welfare systems.

### Managing challenges in complex health and welfare systems

The complexity of health and welfare systems in terms of multilevel perspectives and multiple caregivers requires a systems thinking view to deliver adequate care. Managing the complex structures also requires an understanding of how interactions between caregivers in the overall care system affect the outcome of care quality through various boundary-spanning processes. This is particularly important for persons in need of integrated care involving multiple caregivers [1, 27–29].

Health and welfare could further be seen as complex adaptive systems [30] characterized by interconnected parts that by themselves can adapt to change. The behavior of a complex system will therefore never be fully predictable and, consequently, neither can it be fully controlled [31]. However, it could be directed towards a desirable behavior through simple rules, routines, and a holistic supporting infrastructure enabling development and QI by using strategic, structural, social, cognitive, cultural as well as technical support. This involves providing multi-professional learning arenas; enabling knowledge-creating activities and the development of a shared understanding of goals between professionals and units of care; supporting interactions across care units through boundary-spanning activities; and providing adequate ICT tools. This could for example be achieved by providing best practices [1].

### The need for connecting and coordinating care providers

Mintzberg [32] suggests, maybe somewhat provokingly, that there is no such thing as “a health and welfare system,” but instead only several different and related interdependent interventions that all seek to cure or care. This approach highlights the need for interconnection and coordination between the different care providers. Lord [33] emphasizes in a similar way that care tends to be organized based on the needs of the organization more than the needs of the patient and that it therefore could be discussed if we do or do not have “a health and welfare system.”

The mission of health and welfare systems in general is, however, to meet the needs of the individuals, families, and communities in the society [21]. This means that professionals together with the person in need of care should start by identifying the needs, resources, and preferences of the person as the central user [34, 35]. The core value in the quality of care is generated by the clinical functional units, where the persons in need of care and the providers together create good care [21]. If this is to be possible, efforts are needed to connect and coordinate care providers.

## Rationale

The challenges involved in connecting and coordinating health and welfare providers are common, which is elucidated in this paper. They have been managed within ESTHER for more than two decades by including the active engagement of individual “Esthers” to develop care together with the various care providers. In this study, we explore how ESTHER, through a strong emphasis on the perspective of the person in need of care from more than one provider, developed and became integrated into everyday activities.

### Aim and research questions

This paper aims to contribute to the understanding of how several care providers improved the care for Esther, including the practical implications thereof, by answering the following research questions:

1. What were the incentives behind, and the conditions created for, the development of the ESTHER project?
2. How was it possible to develop the ESTHER project into a mindset and integrate it into everyday activities?

## Methods

This study is a retrospective longitudinal case study with a qualitative mixed-methods approach to receive rich data [36–38] exploring the developmental process of ESTHER. The method included material from individual interviews, focus groups and documents such as project plans, strategic plans, business plans, steering meeting notes, course curriculum, etc. In the mixed method, those data sources were merged and analyzed as one data set [39].

### Informants and selection criteria

The sampling method was purposively [39]. To identify relevant informants for individual interviews, persons from the first steering group of the ESTHER project were asked to assist in identifying key informants. Many relevant persons were already retired, but it was considered important to include them to capture the ideas behind the start of the ESTHER project. Gender balance was intended but assumed to be difficult to attain as most of the staff were females. However, 8 of the 17 informants included were male (see Table 1).

**Table 1:** Informants in the study

				Person with care experience	Staff Hospital	Staff Primary care	Staff Municipal care
Informants	M	F	Age				
Interviews with key informants	3	4	50–70	0	2	2	3
Focus group 1	3	2	40–80	2	2	1	0
Focus group 2	2	3	40–65	2	1	0	2
Total = 17 informants, includes 5 ESTHER coaches	8	9	40–80	4	5	3	5

The inclusion criterium used for informants in individual interviews was that they had to be staff who were actively working at the start of the ESTHER project and were part of the project group. The inclusion criteria for informants in the focus groups were that they had to be staff or persons with different health care needs and at least three years of experience of the ESTHER concept. In both groups, they were to represent different professions from different care providers — hospital, primary, and municipal care — as well as different generations and gender.

All intended informants in the focus groups were individually asked by email if and how they wanted to participate. In line with the intentions of ESTHER, the informants together with the first author decided to perform blended focus group interviews including both staff and persons in need of care. This would be most fruitful as the data collected in this way would benefit from the richness of such a method.

### Data collection

Data were collected through document analyses, individual interviews, and focus groups between May 2020 and August 2021 and covered experiences from 1995 to 2020. In total, seven retrospective individual interviews and two blended focus groups (see Table 1) were conducted by two authors (NV and AA). Document analyses were performed to capture the documented history to back up the interviews.

Relevant documents were identified in local project documentation and web publications. A semi-structured interview guide, developed in the ongoing Canadian study focusing on the international dissemination of ESTHER, was adjusted to the local context and then used to capture the specific local Swedish conditions. The interview guide was pilot tested with one informant, but no adjustments were needed.

Individual interviews with managers and key staff were performed to capture the start of the ESTHER project two decades ago. The individual interviews were followed by blended focus groups with professionals and “Esthers” to trace the development of ESTHER over the last decade. Due to the corona pandemic, the interviews and focus groups were performed digitally or by telephone during the spring of 2020. By then, all the informants had become used to the digital format. Everything was recorded and transcribed verbatim.

### Data analysis

All authors were actively engaged throughout the analysis. Documents were analyzed by identifying key events on a timeline to capture the ESTHER developmental process [40].

The transcribed data were analyzed in accordance with an open inductive thematic analysis [36, 41–43]. The transcriptions were repeatedly analyzed, discussed, and then coded by the authors together. The initial coding was clustered in themes on a web-based dashboard several times in an iterative process. The material was further sorted, subthemes were subsequently identified and grouped into three themes in relation to the aim and research questions. The themes and subthemes were repeatedly reviewed and discussed among all the authors, and the labeling was elaborated on until consensus about the representativeness was established.

All informants were invited to a webinar to comment on the draft results. Six of them, including two “Esthers”, participated. Thereafter all the different data analyses were merged into a final result [36]. To exemplify the results, citations were used, marked in consecutive order (e.g., Interview (I) 1–7 or Focus group (F) 1,2).

### Ethical considerations

This study was part of a larger project carried out at McMaster University, Canada, and Jönköping University and was ethically approved by the Swedish Ethical Review Authority Dnr:2019–04113.

## Results

**Fig. 1:**
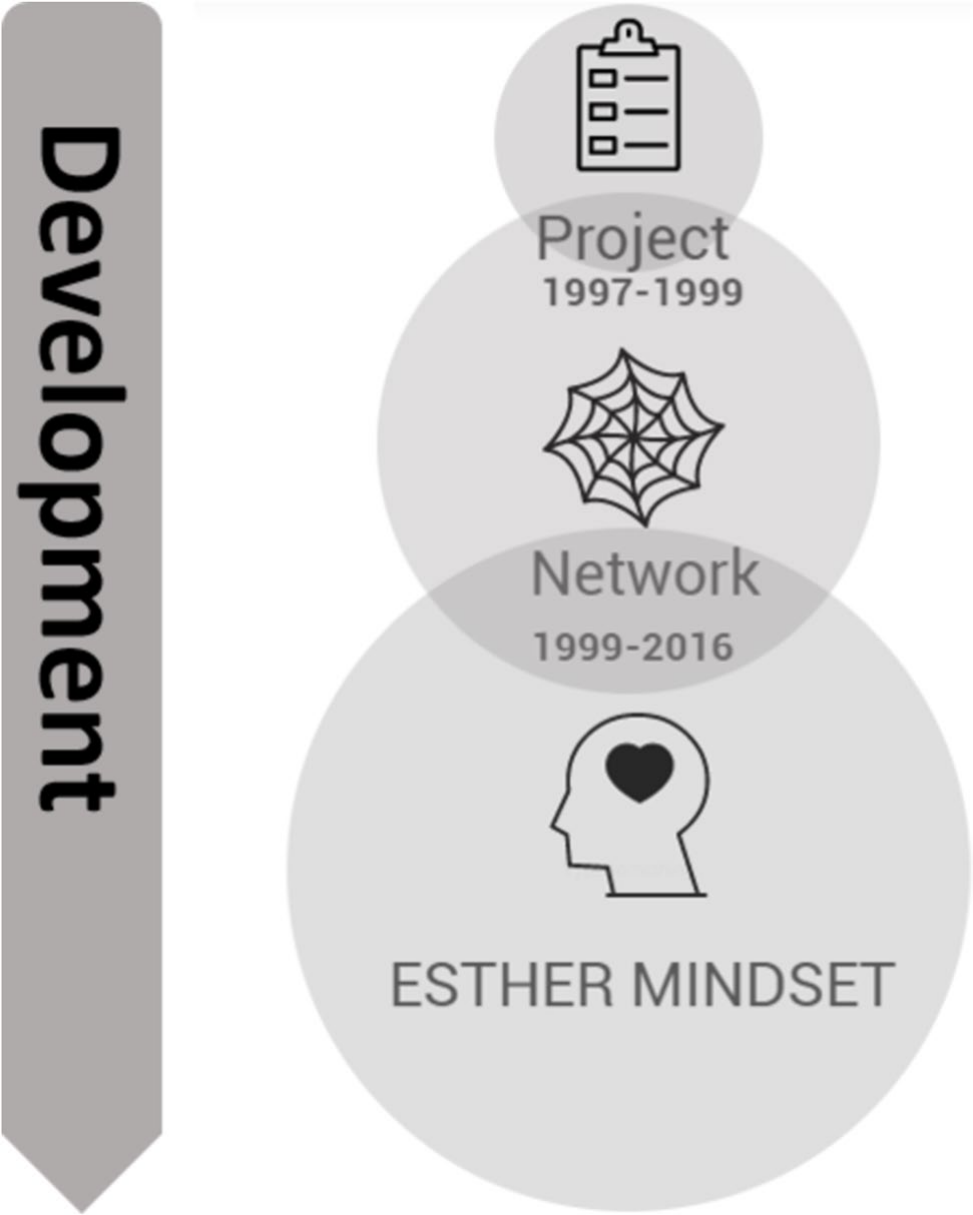
The development of ESTHER from project to mindset

The document analysis showed that the development of ESTHER can be divided into three different periods, partly overlapping, leading to the transformation from a project via network to an ESTHER mindset (Fig. 1). The starting point was a formalized two-year project with a steering committee and a project group that was well established in clinical work. The project participants were multi-professionals from different care providers, which laid the foundation of the network. This constellation was refined during the network period, which lasted for two decades. During the network period, the improvement work was still anchored through a specific ESTHER steering committee. As the ESTHER mindset gradually became more integrated into daily work, routines, and policies, there was no longer a need for a steering committee to lead the work.

**Fig. 2:**
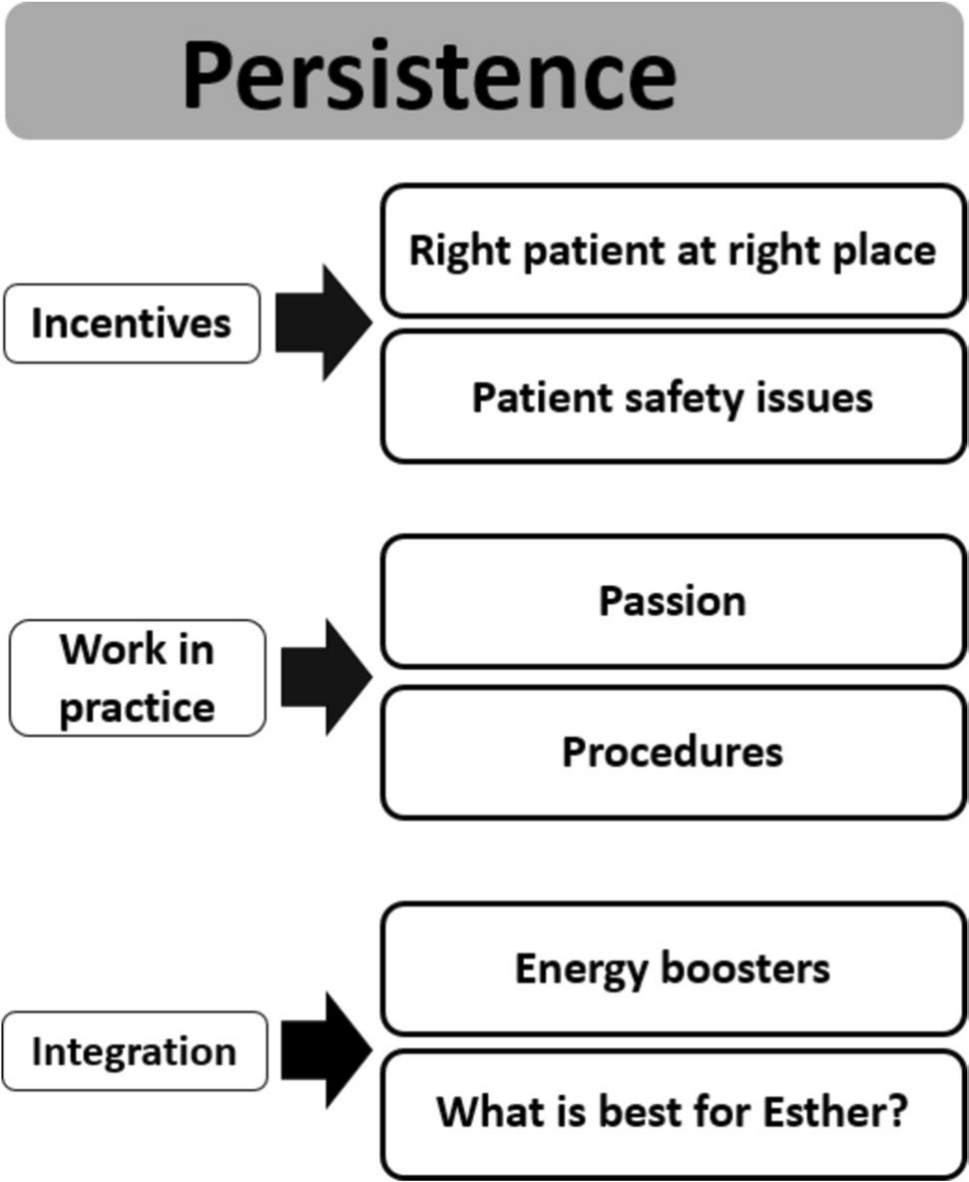
The themes emerging from the analysis of the data

The data resulted in one overarching theme, Persistence — capturing the pervasive spirit as a driving force in the ESTHER mindset. The three themes Incentives, Work in practice, and Integration emerged as characterizing the development of ESTHER. These three themes crystallized out of six subthemes with more specific content related to prominent aspects during the development (Fig. 2).

The development demonstrates persistence, as the ESTHER concept still is an active way of working after more than two decades. While Fig. 1 illustrates the development journey, the themes in Fig. 2 characterize the transformation from project to integration in daily work.

### Incentives behind the project

The first theme, Incentives, consists of the two subthemes *Right patient at the right place* and *Patient safety issues*. The ESTHER project developed in a context of care with long waiting lists, overcrowded hospitals, staff shortages, a diversity of clinics, and primary and home care providers with different documentation in incompatible information and communication systems (ICT-systems). This hindered sufficient information flow and communication between the different care providers within the health and welfare system, which became a patient safety risk. The informants exemplified this with Esther´s prescription lists that showed differences depending on which care unit Esther was visiting. At the time, each care provider had their own way of documenting and thus did not use or did not know what the other collaborators in the care process already had documented.

To solve this problem, a common computer (information) system within the Region Jönköping County was introduced in 2008–2010. As a result, all primary care units and specialist clinics could see the same information about the patient and work in the same prescription list. Parts of the system are now also used for communication and care planning together with the municipalities’ care and nursing.

#### Right patient at the right place

The question “Do we have the right patient at the right place?” was initially raised by professionals from the medical clinic at the Highland Hospital. The project dared to question the need for hospital admission and wondered if care could be delivered in another way by another care provider or unit than specialist care at the hospital. This resulted in the development of visions to work more proactively and prevent hospital admissions. The medical clinic could not create a new way of working on their own. The professionals at the clinic were aware that one caregiver in the local health and welfare system— e.g., hospital care —would influence another caregiver —e.g., home care services —and vice versa. Out of this grew the idea to create joint proactive contributions with the purpose of building a well-functioning local care system that met the needs of the inhabitants through the most appropriate caregiver and unit.

#### Patient safety issues

Besides the challenges noted at the medical clinic, difficulties were identified in the process of prescribing care/treatment and medication at the different care providers. This was stressed by a serious incident in the hospital discharge process. A person was sent home without clear instructions to home care services, which led to interrupted treatment and critical harm for the person. Even though it was just one person, this incident motivated home care providers to engage in better cooperation and information sharing with the hospital, which is illustrated by the following quote from a home care manager during the ESTHER project period:

*My role was to describe what problems we had and what kind of information we needed so that communication could flow well and how we could solve safety problems together. (I 2)*

These were the main incentives highlighted as reasons behind the start of the ESTHER project. It shows a need and willingness to improve care through joint contributions from different care providers. A steering committee of care managers from six local municipalities, the Highland Hospital, and primary care centers in the area was established. Together they applied for funding from the European Social Fund and received a two-year grant for the ESTHER project.

### Work in practice

The second theme, Work in practice, consists of the subthemes *Passion* and *Procedures*. The informants identified this combination of subthemes during both the project and the network period. Passion is about how professionals became motivated and interested in participating in and maintaining ESTHER. Procedures describe the ways of doing things, e.g., the design and content of ESTHER meetings. Procedures are presented through several subheadings to explain the complexity of the subtheme. The informants identified a combination of several methods, structures, and the creation of the right atmosphere that enabled work in practice.

#### Passion

A crucial driving force in ESTHER was to systematically identify and answer the question “What is best for Esther?”. This marked the end of the narrow-minded reasoning of “how can we do better in our department” and instead focused on the person by answering the question “What is best for Esther?” and let that guide how care was to be organized together with other care providers. With this question in mind, professionals became more aware of the importance of cooperating with next of kin and professionals from hospital-, primary-, and municipal care. This new innovative way of thinking nurtured a passion for change and was facilitated by using the Esther story in combination with systematic improvement work. The latter is described more under the subheading Procedures below. The Esther story described in the introduction of this paper was identified by several informants as a very powerful fuel for change as it made sense and motivated professionals to implement a range of improvements. One informant said:

*The fact struck us very quickly that there was great power in the story about one specific person—Esther. (I 4)*

The Esther story was an eye-opener for care professionals in the local health and welfare system. It was found to build an understanding of the care delivery system from Esther’s perspective, and it created a higher awareness of professionals being part of a bigger care system than their specific care unit. At the same time, it triggered an emotional response and cultivated a passion to change for the better for Esther. The story about Esther’s care journey could by the professionals further be associated with their mother or grandmother crossing several organizational boundaries within the health and welfare system. It showed that adequate care only can be delivered if there is a continuity of care and an understanding of how professionals can assist one another and support those who take care of the next step in the care process. The story also helped professionals to create a shared affinity labeled passion in Fig. 2. They felt that it was right and aligned with their inner professional values, as the following quote shows:

*I think it is a basic feeling that we share. We are not here in our professional roles for our own sake. We are here to make the best for our Esthers. I think it's a belief we all share and that we're passionate about. (I 3).*

#### Procedures

This subtheme explains how the work was done, developed, and experienced in practice. It is divided into five further subheadings: 1) Cross-professional forum, 2) The Health Process Reengineering (HPR) method, 3) Learning by doing, 4) Networking, and 5) The ESTHER coaches.

##### Cross-professional forum

As it was clear that there was a need for change and a willingness to make this change together with the different care providers in the local area, it was important to engage representatives from all care providers involved in the project. This was done by a combination of bottom-up and top-down approaches, which means that frontline professionals closest to Esther together with senior managers from different care providers in the ESTHER project faithfully worked together. The frontline staff’s knowledge and skills in real practice were appreciated as a more reliable guiding source than formal guidelines, policies, and other written documents, as they possessed the knowledge and real-life experience of gaps in the care chain between caregivers and how these gaps potentially could be solved. This was expressed in the following way:

*If it [the project] had consisted of just managers, I think we never would have solved that [the problem of care over unit boundaries]. The way to improve was in very practical things. (I 4).*

Several informants explicitly expressed the importance of trusting the knowledge and improvement ideas of the frontline staff and taking those ideas seriously, illustrated by this quote which also highlights that value always is created in the specific moment when Esther meets the care professionals:

*Those who do the work in the [care] process often have the solution. You [manager] have confidence in those who do the work closest to the patient if I say so. The moment of truth [when the person in need of care meets clinical staff] is still there. (I 5).*

A positive consequence of this way of working was the building of quality improvement competence simultaneously at both the frontline and the senior management levels. The informants especially mentioned the atmosphere during all forms of ESTHER meetings, which could be workshops or just planning meetings. They described a very welcoming, open atmosphere where people liked to meet each other over a shared purpose and at the same time took responsibility for their contributions to improve safe care delivery. This was facilitated by creative exercises, daring to think outside the box. The atmosphere was also characterized by curiosity for each other’s work, not blaming but seeking solutions together. The informants stated that a criticizing and blaming culture would not work. The lack of a good atmosphere was described as one reason why other initiatives, trying to copy the ESTHER project, sometimes failed, as specified by the following quote:

They did not find the tone.(I 4)

Another informant said:

*This positive attitude of trying to encourage each other's responsibility and not blame each other was crucial. (I 2)*

These multi-professional and boundary-spanning meetings helped to build mutual understanding and awareness about Esther´s fragmented care and created the possibility of bridging the organizational boundaries between care providers. “Esther should experience us [professionals from different care providers] as one care provider” became a common message. Another comment reflects the development of a joint mission:

*We were no longer “we and them”, we had a common goal, for Esther. We created a strong discussion with the municipality or conversation, you could say — a collaboration that we did not have before. We had worked in parallel tracks which by now became the common image and it was really at that time [1997–2002]* *when it happened quite revolutionary. (I 2).*

The informants further mentioned that professionals need to be trained to take responsibility for their own work as well as support the next provider in the care chain, which cultivated holistic systems thinking.

##### The health process reengineering method

The project was based on the HPR method [37] and other QI methods. The HPR method, introduced by a consultant, was originally adapted from Business Process Reengineering to shift focus from separate parts in the business production system to cohesive processes. HPR was chosen as a method to capture what matters to the person in the care chain process and to develop more patient-oriented (person-centered) care. The HPR method inspired the use of three questions to be asked and analyzed in the following order to keep Esther´s perspective as a guiding star:

1. What is best for Esther?

- The answers led to the identification of Esther´s prioritized values.

2. Who needs to cooperate to get this done?

- The answers identified prioritized stakeholders.

3. What improvements need to be made?

- The answers led to action plans to start improvement projects.

**Fig. 3:**
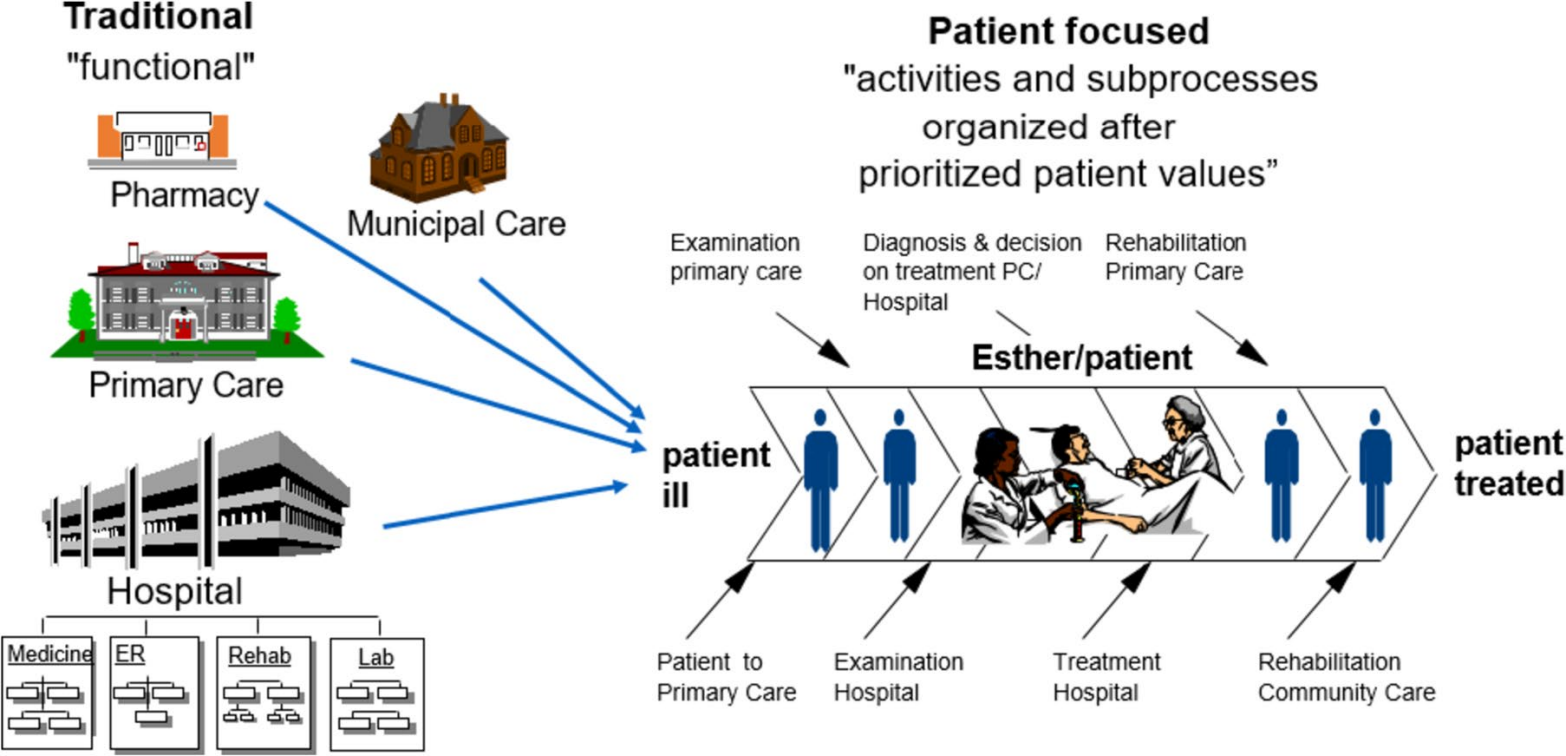
Illustration of the HPR method, developing a process-oriented healthcare system. Source: Region Jönköping County, used with permission [44]

The HPR method is illustrated in Fig. 3 and shows the idea of changing from a traditional function-based to a process-oriented and thereby person-focused care organization. Moving from a functional to a process-oriented healthcare organization requires radical change in the organization and a high level of flexibility from the personnel.

Some of the informants thought that the HPR method was a bit tough, too industrial, and focused too much on efficiency. The Esther story helped as a pedagogical tool to understand the method, as the following quote illustrates:

*For me, it is like this, if you do not use [the] Esther [story] as a metaphor or as a narrative, then I do not think it creates understanding [for a holistic system view]. (I 1)*

The HPR method was further adjusted with the help of focus groups to fit the care context and include soft issues, as described by a participant below:

*We combined this method, HPR, with getting the users in [the Esthers’ preferences and needs]. Focus groups were used to interview different patient groups about how they experienced their care. What was good for the patient, and that way both soft and hard values ​​were considered [to inform what was important to improve]. (I 5).*

Although the method was a bit tricky, the informants mentioned the importance of using HPR to build up an understanding of processes in the local health and welfare system and in the structure of the improvement work.

##### Learning by doing

Talk less, do more, and learn was another lesson highlighted by the informants. They pointed out that focusing on actions, and making changes in the way of working, was an attractive way forward. This was done systematically based on the HPR method and other quality improvement methods. Small tests, using Plan-Do-Study-Act (PDSA) cycles, were promoted by the management. Openness and tolerance toward testing new ideas resulted in continuous improvement work. Starting small improvement projects made the staff proud of their work, which also gave them the energy to continue. The role of the steering group and senior manager responsible for each care provider was described as anchoring the improvement ideas and being supportive. This way of working meant that both major and minor improvement suggestions from professionals were included in creating the yearly action plan of what should be improved. Nevertheless, the informants also pointed out the need for a proper analysis of the care process. Support was provided by managers by scheduling time for improvement work and showing appreciation for the work done by the frontline staff.

##### Networking

After the project time, the name changed to the ESTHER Network and this was extended to several clinics at the Highland Hospital. The network was coordinated by two staff members, one from the hospital and one from municipal care, as a part of their normal job. All professionals involved embraced the twofold motto of “all have to do their job and improve it.” There was no special economic arrangement or remuneration for meetings or improvement work. Department managers were supposed to be very supportive and allow staff to participate in the activities of the network. This worked well at first but became more difficult over time with the involvement of new managers from other care providers who were not used to letting frontline staff take a lead.

The structure of the network was by some informants recognized as a strength and an opportunity to influence improvement work without formal leading positions, exemplified by these words:

*I think ESTHER is a community of professions, it is over organizational care boundaries as well. This is exactly the foundation, gaining both a community and an understanding of the different parts of care /…/. I had never imagined that we [from the municipality] would sit and talk with someone at the hospital and discuss care. It had never crossed my mind before I joined ESTHER that I could have an influence. (F 1).*

Two awards in early 2000 acknowledged the quality of the ESTHER work, and as a result, similar projects popped up in other Swedish regions with different names, e.g., LINNEA in Växjö, HILMA in Örebro, and HELGA in Skellefteå. They called themselves “the cousins of Esther” and all kept in touch to learn from one another. This is still central and helps everyone to develop further. Every year, a conference is arranged and hosted by one of the “cousins”. An example of inspiration that ESTHER gained from the HILMA project stems from the HILMA ambassadors training, a way to introduce new staff to a new mindset, which planted the idea to develop so-called “ESTHER coaches”.

##### The Esther coaches

The steering group of the ESTHER Network decided to develop local ESTHER coaches with the ambition to keep momentum and further develop the network by retaining a focus on what is best for Esther and increasing the capability to make necessary changes to local care units and care chain processes. This was funded by a new grant from the council of the European Social Fund in Sweden. Region Jönköping County in cooperation with its municipalities started to educate ESTHER improvement coaches in 2006. As this was a completely new role in health and welfare, the participants were encouraged to reflect on and contribute to the development of this role. A lot of work was done to differentiate an ESTHER improvement coach from any other improvement coaches. ESTHER coaches were professionals from different disciplinary backgrounds and a range of care providers in hospital-, primary-, and municipal care. Most of the ESTHER coaches worked at the frontline of services close to the person in need of complex care (Esther), and some were managers. The coaching of various improvement projects became part of their daily work alongside their work as a professional nurse, social worker, or other care professional. They always made sure that all the improvement work benefited Esther as well as the health and welfare organization.

### Integration

The third theme, Integration, regards how ESTHER during the network period became integrated into daily activities with less formal structure, as more of a mindset that was enabled in practice by the ESTHER coaches and anchored in Jönköpings county health and welfare policies and business plan. The informants described the way of working as exciting, creative, meaningful, and structured, which cultivated persisting commitment. However, commitment also needs energy to flourish. The informants identified specific elements, mentioned as energy boosters, that helped to support integration and encourage persistence. Further, integration was promoted as the question “What is best for Esther? “ was used to set the tone in all cooperation with different care providers.

#### Energy boosters

Energy boosters are presented in two further subheadings: Awards and rituals and Co-production.

##### Awards and rituals

The energy was primarily found in an intrinsic motivational drive to provide better care for Esther, described earlier as “Passion”. This passion was boosted by awards and acknowledgments. ESTHER was acknowledged by CNN in 2014 as “one of the coolest innovations around the world,” and was recognized in 2017 by the European Prize for Social Innovation in the area of active and healthy aging, which inspired other health care organizations. As a result, visitors came regularly from all over the globe to understand and learn more about ESTHER in the Region Jönköping County. Professionals and “Esthers” presented their work to the visitors and received new questions about the concept. It made them feel proud of their work, as the following quote shows, expressed by three different informants:

*I have been very proud to be a part of ESTHER! (I 1, I 4, I 7)*

Another energy booster was the yearly celebration of Esther’s name day, initiated by the ESTHER coaches, as described below:

*We celebrate our name day (ESTHER) with a cake or something, but the ESTHER coaches were not content with baking cakes in a nursing home or that the hospital kitchen sent up a pastry. So, they spoke to and invited local cafes to participate in this and that's something the cafes after a few years did spontaneously. They announced, started manufacturing and selling ESTHER pastries on March 31. (F 1).*

The coaches also introduced the ESTHER flag. The ESTHER flag is red with the text “Is this best for Esther?”. It was used as a symbol and a reminder to be alert, take a moment of reflection, and go back to the basics — are we trying to improve for the best for Esther, or what? The coaches started to use these red flags and nowadays the flags are seen all over the Region Jönköping County, used by department managers, professionals, and “Esthers” themselves.

##### Co-production

The ESTHER coaches specifically recognized that new energy came about by actively involving and engaging “Esthers” themselves in the meetings and in the improvement work. The coaches together with “Esthers” became the agents of change for this new development, which today is called co-production. Besides the new energy that emerged, this made sure to prevent wrong assumptions about what is important to Esther and generated clever improvement ideas. This is illustrated by the following quote:

*The patients’ stories, by far the most valuable. Absolutely. Because that is exactly what is valuable to patients. And I discovered this early in my career—that we can believe and think that this and that is important for the patient. But if we ask them and get them to tell us what is most important to them, they say completely different things. And if they get help with it, everything else is solved. That's how it is. I have been through it several times. We are completely wrong many times. We (professionals) think we know, but we do not. (I 2).*

The informants in the focus groups especially named two “Esthers” who played a big role in developing ESTHER toward more co-production. Their attendance was important as they always asked: “In what way has this discussion helped me? Is care getting better by talking about it?” They encouraged staff to act in the nearest future (next week). These questions became standard in workshops and meetings in co-production with persons in need of care. It also became natural to always have several “Esthers” in the room when talking about how to improve care, which affected the staff’s perception:

*We believe in our Esthers, our customers, our patients, whatever term we use. Not only do we believe in their ability to express their needs but that it helps both them and us. (I 3).*

#### What is best for Esther?

Professional awareness of the interdependency between different care providers grew stronger and a more holistic mindset was developed based on the question “What is best for Esther?”. This became clear during a large organizational change in 2016 when the three hospitals, located in different areas in the region, were reorganized into three Divisions of Care. This led to an extension of the care providers involved in Esther’s care, which came to include 3 hospitals, 13 municipalities, and 46 primary care units. To always incorporate what matters to Esther, the person in need of care, ESTHER became a formalized trademark simply because it focused on a collaborative way of working to improve care with and for Esther in the whole Jönköping region. This mindset became a part of all leadership education in the Region Jönköping County.

The mindset is not about competing but rather about complementing and supporting one another to create the best possible outcome for Esther, expressed like this by one of the informants:

*I actually think that we in the region have taken this to heart, the lessons we received from ESTHER and all work is based on that concept, in all our projects. (F 1)*

## Discussion

This study shows how co-production and person-centeredness through a well-established QI strategy could improve care for persons with multiple care needs involving more than one care provider —”Esthers” — and shows what conditions made it possible to permanently integrate the concept in daily operations after the project phase. The knowledge platform of QI is usually described through four dimensions: systems thinking, the psychology of change, learning, and understanding variation [21, 45]. Three of these dimensions, namely systems thinking, psychology of change, and learning, were identified as important drivers in supporting the transformation from a project to an ESTHER mindset. The fourth dimension, which is about measuring and understanding variation, was developed later. Today, professionals in Jönköping County are extremely careful about following all data over time and discussing variation.

The three QI dimensions are further discussed below, addressing their influence in practice, the influence of contextual conditions, and practical implications for other health and welfare organizations wanting to develop a similar concept.

### Promoting systems thinking

The simple question “What is best for Esther?” opened the door for systems thinking despite the different actors organizationally belonging to different caregivers. Thinking about the system as a whole differs from the common way of imagining health and welfare organizations as linear and hierarchical, and requires an open mindset [46]. Creating meeting places over organizational boundaries turned out to be an investment that facilitated the creation of an open mindset. Without this kind of meeting arena, it is very difficult to understand how the work in one unit influences another, particularly regarding other care providers.

Palmberg Broryd [31] suggests that leading complex systems works better with simple rules and attractors than detailed routines. By frequently using the straightforward question “What is best for Esther?” it was possible to focus on the most essential thing in daily work, namely what matters to the person in need of care. The question was used across functional units and across caregiver organizations, which nurtured task-aligned changes and systems thinking. The question “What is best for Esther?” is still relevant in the Region Jönköping County. This was shown, for example, by the fact that during the corona pandemic which dominated healthcare in recent years, the Region Jönköping County held information meetings with all managers every week. Those meetings always ended with the senior management presenting three simple rules:

- Best for Esther,
- Take responsibility for your step in the care chain—give feedback to the step before and facilitate the next step,
- We manage together.

### Paying attention to the psychology of change

The psychology of change is described as the human side of change and has its roots in psychology and change management. It includes understanding how people react to change and how to achieve commitment [22, 47]. The ESTHER transformation from a project to a mindset can be understood as a combination of methods, with attention to the psychology of change clearly embedded, including a unique combination of constantly stimulating and boosting intrinsic staff motivation (e.g., through the Esther story and by celebrating the name day). A more conventional way of stimulating motivation is the so-called “pay-for-performance”, where care units get extra pay for desirable work processes and outcomes [48, 49]. This was never the case in ESTHER. The improvement work was framed as part of the regular daily work. Research has shown that this kind of integration is crucial for building perseverance [26]. The results demonstrate the possibility of shaping long-term commitment by nurturing intrinsic motivation, described as “passion” by the informants. The theory of self-determination supports that intrinsic motivation is key to building perseverance [50].

As ESTHER resulted in improved processes that reduced the duplication of work and increased patient safety, both employees and the care provider organizations experienced benefits. The organizations were able to use resources in a more efficient way, including more efficient administrative routines in care planning and documentation, which led to nurses having more time to spend with Esther.

Procedures that supported the collaboration between care providers were found to be crucial, which is in line with person-centeredness [15, 16, 18]. The atmosphere established during this collaboration process was also important. The creation of an open and warm atmosphere where people feel psychologically safe is crucial to building commitment for improvement work in the long run. Nembhard [51] stated that creating psychological safety, where people feel free to speak up without being humiliated, is a key ingredient to building high performing teams and can predict engagement in improvement work. To our knowledge, the psychological atmosphere is seldom described in reporting improvement work. We propose that it is an important factor that should be recognized and described more in QI literature as part of the psychology of change.

Another reason for paying more attention to change psychology is that many initiatives for change fail today, and staff often experience “project tiredness” [52] as they do not experience any concrete results of a change project or simply are not informed about it [43]. Perhaps QI initiatives favor methods at the expense of paying attention to the psychology of change. Focusing on methods alone can be experienced as too technical, as mentioned in this study. Without the Esther story — another human aspect of change — staff would not have been passionate about improving, as passion is based on emotions related to specific situations that staff can identify with, and not just methods. This study confirms that to facilitate long-term commitment, it is necessary to pay attention to the human side of change, which is also stated by Bate et al. [29].

### Embracing co-produced development and learning

Successful change builds on the development of a shared vision and commitment to change [1, 53]. In ESTHER, this was cultivated by constantly highlighting the perspective of the person in need of care—Esther. It also created a desire to learn how to make care better and safer for Esther. The Esther story was important from the very beginning, and professionals were guided by the question “What is best for Esther?” However, over the years, the co-production view became stronger [24, 34, 35], and Esther herself was invited to actively cooperate in the improvement work. This could be seen as a natural development, although it is not easy to practice in a complex health and welfare system. Nevertheless, who else than Esther herself can answer the question “What is best for Esther?” This is fully in line with person-centered care in the sense of inviting and involving the individual in their own care [19]. As shown by Cooney and O’Shea [20], a narrative such as the story of Esther can be a powerful pedagogic tool to facilitate person-centeredness and initiate partnerships to co-produce care, which also was prominent in this study. A clear narrative makes it easier to look at problems, focusing on action and allows power to be shared.

As health and welfare systems by tradition are hierarchically structured and span across organizational boundaries, creating real partnerships built upon a foundation of shared power and trust is a challenge [46]. In this study shared power, as the possibility to influence improvement work, was an important driver. We found that power was shared between different groups, not only between different care providers, managers, and frontline staff, but also between professionals and persons in need of care. The omnipresent question “What is best for Esther?” facilitated dialogues, focusing more on the common goal and less on hierarchy and specific caregivers. This was emphasized further as persons in need of care were actively engaged in co-producing the development of ESTHER. The skills and routines of co-production were developed in practice by staff and persons in need of care and became a natural way of working in ESTHER [24]. However, there is still improvement potential when it comes to redefining power sharing through constant reflection and action regarding power dynamics [54].

Another enabling factor when it comes to learning was “Attraction by action,” which emerged through the learning-by-doing approach. The focus on action attracted staff to participate as it entailed a direct difference for Esther. This is advocated in QI methods by performing small PDSA cycles and learning from them [22, 45]. Likewise, Beer et al. [53] argue that effective change starts with task alignment, where the primary goal is to change the way of working instead of trying to start by changing individuals. Effective change is also strengthened by creating a long-term capacity for continuous learning [53].

In the beginning of the ESTHER project, a consultant introduced the HPR method. Over the years, however, the teaching role was taken on by individuals within the local regional care system, serving to foster consensus for the vision and build long-term capacity and improvement capability within the system. This is a crucial factor for integrating a project into daily work procedures as it is important to make the staff experience that they own and can influence the process. One way of doing this was creating the ESTHER coaches, recruited from different caregivers and organizations, who all got the same education.

### The importance of contextual conditions

This study was performed in a regional health care context that for decades had continuity in both political and county council leadership embracing QI as a business strategy, with a long-term commitment to alignment from macro to micro level [55]. This attitude, which encompassed the whole region, made it possible to co-ordinate and establish appropriate and creative meeting forums without having to ask for permission from senior managers. This managerial context to a high extent contributed to making ESTHER successful, since staff experienced it as dynamic and possible to influence. We conclude that the cultural aspect, where professionals did not wait for permission and clear task descriptions from senior managers but started small improvement projects as a part of their daily work, has promoted the success of ESTHER. Likewise, the recognition and support of improvement work from senior managers was crucial.

Pre-conditions like these can also be cultivated by senior managers and leaders in other contexts to support initiatives from the frontline staff. The informants in this study talked about relentlessly building and changing their own context, testing new ways of caregiving, guided by the basic question “What is best for Esther?” That is only possible in a tolerant context where managers trust professionals.

### Practical implications

The study is conducted in a local regional Swedish context. To become even more widely applicable, ESTHER will be further tested, refined, and described in different international settings by ongoing follow-up studies. We believe that this study captures overarching as well as specific fundamental insights that can be transferable to other health and welfare settings, while details might need to be adjusted. This study also highlights the importance of combining QI dimensions as a foundation for perseverance [56].

### Conclusions

The perseverance of the ESTHER concept was shaped by a simple question, “What is best for Esther?”, that never changed. This question unified people, flattened the hierarchy, and gave direction for a new way of working and a new mindset, always centered around Esther. The work flourished as it mobilized the people who know the most — frontline staff and persons in need of care — within a permissive leadership environment where “Quality as a strategy” was a keystone. This was strengthened by using QI methods and engaging Esther herself in the QI process. The education of professional ESTHER coaches as driving forces stabilized and kept the concept alive after the project phase. By organizing a network between caregivers anchored in the health and welfare region’s different policies and business plans, it was possible to further strengthen the ESTHER mindset.

The main lessons learned can be summarized as follows:

- Develop the cornerstones of QI both as a strategy and in practical application.
- Focus on the core objective, namely the care recipient’s needs.
- Create informative multidisciplinary and boundary-spanning dialogues.
- Encourage integration into daily work through tailored infrastructure and continued support from senior managers.
- Invent new forms of support functions in line with what long-lasting integration requires.

### Strengths and limitations

This case study captures retrospective data of twenty years of development. Using a mixed methods approach including documents, individual interviews, and focus groups, gave a rich data material. Baker [38] asserts that case studies are particularly helpful for making sense of complex relationships in healthcare and can contribute to knowledge on how to improve care. All authors were deeply involved throughout the analysis to make sure that the close relations that some of the authors had to the project would not interfere.

At the same time, the knowledge these authors had and their long-term engagement in ESTHER were preconditions for the study. To strengthen the study, the informants validated the results through an inter-reliability check, which increased the trustworthiness [57]. The informants had influence on the composition of the focus groups and they all agreed that the groups should be blended, which also is in line with the ESTHER mindset and thus further strengthens the focus of the Esther concept. The close interaction between researchers and practitioners enabled mutual learning and the immediate use of new findings locally [58], which increased the usefulness of the study results [59].

## Data Availability

Data is available on request to the first author (NV) on an aggregated level due to confidentiality and GDPR regulations.
